# Recovery of Ag(I) from Wastewater by Adsorption: Status and Challenges

**DOI:** 10.3390/toxics12050351

**Published:** 2024-05-10

**Authors:** Qiang Wang, Mengling Li, Meng Xi, Mengyuan Zhao, Xiaotong Wang, Xiaoyu Chen, Lin Ding

**Affiliations:** 1 Key Laboratory of Jiangxi Province for Persistent Pollutants Prevention Control and Resource Reuse, Nanchang Hangkong University, Nanchang 330063, China; 2 National−Local Joint Engineering Research Center of Heavy Metals Pollutants Control and Resource Utilization, Nanchang Hangkong University, Nanchang 330063, China

**Keywords:** adsorbents, Ag(I), adsorption mechanism, adsorbent optimization

## Abstract

Untreated or inadequately treated silver−containing wastewater may pose adverse effects on hu−man health and the ecological environment. Currently, significant progress has been made in the treatment of Ag(I) in wastewater using adsorption methods, with adsorbents playing a pivotal role in this process. This paper provides a systematic review of various adsorbents for the recovery and treatment of Ag(I) in wastewater, including MOFs, COFs, transition metal sulfides, metal oxides, biomass materials, and other polymeric materials. The adsorption mechanisms of these materials for Ag(I) are elaborated upon, along with the challenges currently faced. Furthermore, insights into optimizing adsorbents and developing novel adsorbents are proposed in this study.

## 1. Introduction

Silver, as a transitional element, holds significance as one of the essential precious metals, primarily found in silver ores and predominantly existing in compound forms [1]. Owing to its distinctive physical properties and chemical stability, silver finds extensive applications across various industries including medical [2], electronics [3], electroplating [4], consumer goods manufacturing, and photography [5]. The principal utilizations of silver are predominantly grounded in industrial, photographic, and jewelry/silverware domains, accounting for approximately 85% of silver demand. According to the World Silver Council report, with the global economy’s recovery growth and the alleviation of supply chain disruptions caused by COVID-19, last year witnessed a substantial surge in total silver demand, escalating by 18% to 1242 million ounces, marking the highest level since 2010. However, due to the rapid increase in silver demand and its scarcity, silver production falls short of meeting requirements. The production of native silver ores remained essentially stagnant compared to the same period last year, experiencing a mere 0.1% increase [6].

Confronted with the rising demand for silver−containing products, improper silver usage not only leads to resource wastage but also inevitably results in environmental leakage during its utilization, particularly generating significant amounts of heavy metal ion−containing wastewater in industrial applications. Accumulation of silver ions in aquatic environments induces toxicity in aquatic organisms, disrupts aquatic ecosystems, and poses potential risks to human health, particularly through food chain transfer [7]. Excessive intake of Ag(I) combines with proteins and amino acids on cell membranes within the human body, diffusing systemically and causing damage to the liver and kidneys [7,8]. Additionally, Ag(I) deactivates thiol enzymes, binds to functional groups on various metabolites, and displaces essential metal ions within the human body [9]. The biological toxicity of monovalent silver even surpasses that of Hg^2+^ and Cu^2+^, as evidenced by the USA Environmental Protection Agency (USA EPA) setting the maximum level of silver in drinking water at 0.1 mg/L (0.1 ppm) [10]. Thus, from an environmental and resource perspective, exploring silver extraction from wastewater is crucial and necessary, not only for reducing excessive exploitation of natural resources but also for lowering production costs, achieving a balance between environmental protection and economic benefits.

Presently, methods for recovering silver ions from wastewater, such as biological treatment [11] and electrochemical methods [12], have made some progress in silver ion recovery. However, they still face challenges such as high costs, lengthy processing cycles, and potential secondary pollution issues [13]. Adsorption methods have gained significant attention in recent years due to their low energy consumption, simple process, convenient operation, and absence of secondary pollution [14,15]. However, traditional adsorbents such as resins, chitosan, and cellulose suffer from drawbacks such as insufficient specific binding sites, restricted mass transfer within pores, and single−mode action, leading to low adsorption capacity, poor separation selectivity, and inadequate material regeneration. Selective recovery and enrichment of silver from complex water bodies remain challenging [16,17,18,19]. Therefore, the development of novel adsorbent materials serves as the foundation and key to achieving efficient adsorption recovery technologies. With the advancement of materials chemistry globally, various new adsorbents have been applied to recover Ag(I) from wastewater, such as metal-organic frameworks [20], covalent–organic frameworks [4], and layered double hydroxides [21]. Their high specific surface area, porosity, and abundant functional groups play crucial roles in achieving selective separation and directed recovery of Ag(I). Despite the significant advantages of these adsorbents, there is currently a lack of systematic research on the adsorption mechanisms and inherent limitations of these adsorbents.

Therefore, this study provides a summary of various novel adsorbent types, their adsorption performance, and mechanisms. It also discusses the challenges faced during adsorption recovery. Finally, new insights are proposed to address the limitations of current recovery methods, aiming to provide a more feasible path for advancing more efficient and sustainable silver ion recovery technologies from wastewater, thereby promoting environmental sustainability and effective resource management.

## 2. Types of Adsorbents

### 2.1. MOFs and COFs

**Figure 1:**
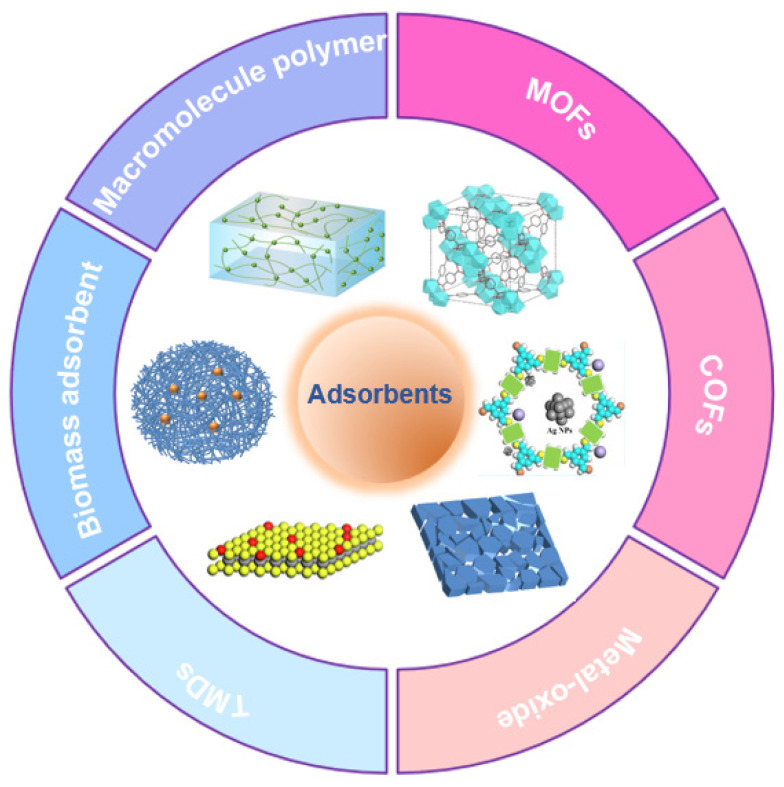
Different adsorbents for the adsorption of Ag(I).

As shown in Figure 1, a variety of adsorbents have been developed for the recovery of silver from wastewater. Firstly, metal-organic frameworks (MOFs) are a highly regarded class of novel porous materials, renowned for their excellent crystallinity, diverse structures, large surface area, outstanding adsorption performance, and tunable functional components [22,23,24]. In recent years, the design of MOF−based adsorbents and their applications in selectively adsorbing heavy metal ions from water have gained widespread attention. According to the HSAB (Hard-Soft-Acid-Base) theory, interactions between hard Lewis acids and bases or soft Lewis acids and bases are much stronger than interactions between hard acids and soft bases or soft acids and hard bases [20]. Therefore, MOFs with enhanced water stability can be obtained by choosing ligands based on carboxylic acid salts (hard Lewis bases) and high−valent metal ions (hard Lewis acids), for instance, mesoporous aluminum−based metal-organic framework materials (MIL−100(Al)), titanium−based metal-organic framework materials (MIL−125), and zirconium−based metal-organic framework materials (UiO−66), among others. Among these, UiO−66, a class of zirconium−based MOFs, has attracted significant attention in recent years due to its highly tunable nature, making it widely applicable for heavy metal recovery in wastewater. It maintains a stable framework structure even in complex solutions like acidic environments [25]. Ding et al. [20] combined rhodanine onto UiO−66 using two modification methods, resulting in UiO−66−Rd_p−m_ and UiO−66−Rd_i−s_. Both materials exhibited superior adsorption performance with capacities of 120 mg·g^−1^ and 109 mg·g^−1^, respectively, showing an approximately sixfold increase compared to unmodified UiO−66. UiO−66−Rd_p−m_, with its highly exposed rhodanine groups, demonstrated rapid kinetic adsorption and outstanding selectivity, achieving efficient capture of silver ions in wastewater. Additionally, after rinsing with a 0.01 M HNO_3_ solution containing 0.1% thiourea, UiO−66−Rd_p−m_ can be easily regenerated and utilized for the subsequent capture of Ag(I). Moreover, UiO−66−Rd_p−m_ exhibits nearly complete removal efficiency for Ag(I) at a concentration of 10 mg/L in simulated lakes, rivers, and wastewaters, further highlighting its promising application prospects.

In addition to Zr−based MOFs, some MOFs can be combined with polymers to enhance material stability, recyclability, and selectivity while retaining superior adsorption performance. For instance, Xue et al. [26] used supercritical carbon dioxide to composite iron−based metal-organic framework material (MIL−127) with poly−o−phenylenediamine. This new method, compared to traditional impregnation methods, increased the adsorption capacity for silver ions, with MIL−127/26PoPD reaching a capacity of 560 mg/g. In the regeneration experiment, 50 mg of MIL−127/26P_O_PD was exposed to a 20 mL solution of Yangtze River water containing 10 ppm Ag(I) for 5 min. Removal rates exceeding 95% were achieved for the first five cycles. However, due to the oxidation of the oligomer, the removal efficiency dropped to 70% in the tenth cycle. To restore the nitrogen−containing functional groups to their original state, the composite material was treated with 0.002 M L−ascorbic acid after every ten cycles. This resulted in an increase in silver removal efficiency to over 95%, demonstrating excellent regeneration and reusability performance. Furthermore, the adsorbent material exhibited excellent antibacterial properties, proving lethal to both Gram−negative and Gram−positive bacteria, thus serving a dual purpose.

In comparison to MOFs, covalent–organic frameworks (COFs), consisting of light elements (e.g., H, B, C, N, and O) connected by strong covalent bonds, exhibit advantages in water stability. Their robust framework, exceptional structural regularity, highly ordered pore sizes, inherent porosity, and chemically stable design characteristics facilitate the uniform diffusion and effective collection of metal ions in wastewater [27]. For example, Pan et al. [4] synthesized COF−SH rich in thiol groups using a simple solvent−thermal method, displaying high selectivity for Ag(I) with an adsorption capacity of 609.89 mg/g. Additionally, to assess its desorption capability, 4.0 mg of COF−SH−Ag was added to 20 mL of various eluents. It was found that the desorption efficiency of Ag(I) using thiourea alone as the desorbent was not remarkable; however, in contrast, a combination of 1–3 M HNO_3_ and 0.3 M TU achieved a desorption efficiency of 100%. Qiu et al. [28] prepared a novel cationic covalent—organic framework (COF−HNU27) by reversible condensation of 1,3,5−trimethoxy−2,4,6−trimethylbenzene (TFBOMe) and ethylene dibromide (EB). This COF exhibited a large pore size, ultra−high chemical stability, and efficiently and selectively captured Ag(I) from aqueous solutions with a high adsorption capacity of 349.6 mg/g, reaching equilibrium within 2 min. Moreover, the material can be easily regenerated in 3 M HNO_3_ and 1 M sodium thiosulfate solution, with the adsorption efficiency retaining 95% of its original capacity even after 5 cycles.

Furthermore, the ability of adsorbents to detect Ag(I) in water is crucial for the adsorption and recovery of Ag(I). Zhang and colleagues [29] employed Tris(4−aminophenyl) amine and 2,2′−Bithiophene−5,5′−dicarboxaldehyde as co−monomers to fabricate a two−dimensional COF based on thiophene under solvothermal conditions (TAPA−BTDC). This COF exhibits high crystallinity and achieves a maximum adsorption capacity of 353.19 mg/g for Ag(I) through intricate structural design and pore control. Subsequently, using a 0.5 mol/L thiourea and 2.0 mol/L HCl solution as the eluent, the material retains 93% of its original adsorption capacity after five cycles. Even at concentrations as low as 2.4 × 10^−4^ mmol/L of Ag(I), it produced a strong fluorescence emission.

### 2.2. Transition Metal Dichalcogenides (TMDs) and Metal Oxides

Transition Metal Dichalcogenides (TMDs) possess abundant surface active sites and tunable structures, with sulfur functional groups on their surfaces exhibiting strong affinity for heavy metal ions [30]. Among them, molybdenum disulfide (MoS_2_) is a representative example. Its layered structure consists of alternating arrangements of sulfur and molybdenum atoms, forming a graphite−like sheet where molybdenum atoms coordinate with sulfur atoms in an octahedral manner. Additionally, MoS_2_ exhibits excellent electronic transport properties in its monolayer form, making it widely applied in electronic devices [31]. However, layered transition metal dichalcogenides may dissolve or undergo structural changes in humid environments [32]. Although MoS_2_ has a large surface area, its original form tends to aggregate in aqueous solutions, leading to poor adsorption performance and water stability. To address this issue, Yang et al. [31] synthesized MoS_2_/MWCNTs nanohybrids using industrially produced carboxyl−functionalized multi−walled carbon nanotubes (MWCNTs−COOH) as a template, and low−cost ammonium molybdate and thiourea as molybdenum and sulfur sources, respectively, via a one−step solvothermal method. This approach addressed the issues of poor dispersibility and low water stability. The specific surface area of the MoS_2_/MWCNTs nanohybrids was 4.5 times that of pure MoS_2_ and the maximum adsorption capacity for Ag(I) achieved was 601.97 mg/g.

Among various water treatment agents, metal oxides demonstrate excellent performance in removing heavy metal ions from water due to their cost−effectiveness, simple preparation, environmental friendliness, and controllable regenerability [33]. Current research indicates that many metal oxides can effectively remove toxic metal ions, exhibiting high capacity and selectivity [34]. Silicon dioxide, with its layered and rigid structure, can easily treat heavy metals in wastewater and withstand multiple −adsorption–desorption cycles [35]. However, despite its large surface area, silicon dioxide’s inert surface and limited functional groups hinder its affinity for silver. Therefore, Mao et al. [36] introduced dual functional groups on the surface of silicon dioxide, preparing a series of imidazole−derived functionalized organic silicon nanosheets (MI, MBI, and MTT) via covalent grafting. Through synergistic effects, these materials selectively remove Ag(I) from wastewater, with maximum adsorption capacities of 70.3, 103.2, and 139.5 mg/g, respectively. Subsequently, 0.1 mol/L HNO_3_ was chosen as the eluent, and after four cycles, the adsorption performance remained superior, demonstrating excellent regeneration capability. In addition to silicon dioxide, manganese dioxide has also been studied for the adsorption of silver in water. Hassan et al. [37] oxidized divalent manganese ions with ammonium persulfate to prepare a cactus−like hydrated manganese dioxide nanocluster, which was utilized for adsorbing Ag(I) from wastewater, achieving a maximum adsorption capacity of 81.97 mg/g according to the adsorption model fitting. In the regeneration experiment, 50 mg of the adsorbent material adsorbed 50 ppm/L of Ag(I) from a 50 mL solution. After collecting the HMDNC particles, the adsorbed ions were recovered using a composite eluent of 3 M HCl and 8%wt thiourea. Even after five repetitions, the removal efficiency still reached 70%.

### 2.3. Biomass Adsorbents

To enhance resource utilization efficiency, renewable biomass materials serve as effective wastewater treatment agents. Biomass adsorbents are diverse, including chitosan, cellulose, and biochar materials. Importantly, compared to traditional chemical adsorbents, biomass materials align better with the concept of sustainability.

Chitosan, regarded as a valuable adsorbent material due to its abundant hydroxyl and amino functional groups, biocompatibility, eco−friendliness, and extensive application prospects, is being extensively studied [38]. However, pristine chitosan powder tends to swell, break, and form colloid suspensions in water, complicating −solid–liquid separation [39]. Recent studies have found that chitosan−based adsorbents can be designed in various forms such as membranes, beads, fibers, and capsules to enhance adsorption kinetics and address −solid–liquid separation issues [40]. For instance, Liu et al. [41] synthesized chitosan beads (CB−G3) with a diameter of 1.40 mm by repeated grafting of maleic anhydride (MAH) and tetraethylene pentamine (TEPA). The abundant N−functional groups on the surface of PAMAM dendrimer shell provided optimal coordination sites for the binding of Ag(I), with a maximum adsorption capacity of 105.62 mg/g. Additionally, the spherical structure of chitosan facilitates the chemical grafting of PAMAM dendritic polymer chains onto the surface of CB. Simultaneously, the adsorbed material can be easily collected through simple filtration, greatly facilitating the recovery of the adsorbent and preventing secondary pollution. After five −adsorption–desorption cycles using 0.05 mol/L HNO_3_, some functional groups of CB−G3 are lost due to acid cleavage, leading to a gradual decrease in its effectiveness towards metal ions, albeit retaining over 70% adsorption efficiency.

Cellulose−based adsorbents also exhibit strong adsorption capacity for target ions in heavy metal adsorption fields and consist of a large number of hydroxyl groups, making cellulose one of the most abundant biopolymers in nature [42]. Biswas et al. [43] modified cellulose with disulfide aminoformate to form a novel adsorbent material, DMC, which selectively captured silver iodide from complex aqueous matrices. Within 60 min, the maximum adsorption capacity can reach 10.97 mmol/g. After washing the metal−loaded DMC with 3.0 mol·L^−1^ HNO_3_, elemental silver can be directly obtained from wet industrial sediment leachate containing 1945 μmol/L Ag(I) solely through incineration.

In addition to chitosan and cellulose, the abundant carbon content in biomass provides a solid foundation for the preparation of biochar materials, which have shown remarkable potential in the field of water treatment. For example, Fan et al. [44] utilized discarded passion fruit peels as a precursor for biomass and synthesized sulfur−doped porous carbon materials (SPCs) through hydrothermal carbonization coupling. Experimental results demonstrated its excellent adsorption of Ag(I) in acidic aqueous solutions, with a maximum adsorption capacity of 115 mg/g. The material also exhibits good selectivity towards various competing cations. Due to the high affinity between the surface sulfur functional groups and Ag(I), even higher concentrations of HNO_3_ solution cannot fully desorb silver. Therefore, employing a simple and effective calcination process for silver recovery proves to be a viable approach. Silver powder can be obtained by calcination at 500 °C for 90 min in a muffle furnace.

To more intuitively illustrate the adsorption and recovery processes of the different adsorbents for Ag(I) mentioned above, a summary is presented in Table 1.

### 2.4. Other Macromolecule Polymer

Polymeric materials typically comprise large molecular compounds formed by the chemical bonding of numerous identical or similar monomeric units, possessing hydrophobicity, large surface areas, and good flexibility. Moreover, the structure and properties of polymeric materials can be controlled by adjusting synthesis conditions, facilitating easier selective capture of heavy metal ions [45]. Among these, hydrogels represent highly hydrophilic three−dimensional network structures formed through specific chemical and physical cross−linking, capable of swelling significantly in water without dissolution. Additionally, with increasing water content, their volume and pore size also enlarge [46]. As early as 2014, Firlak et al. [47] fabricated a novel thiol−ene−based hydrogel P(Penta3MP4/PEGDA/AAc) using UV−curing technology. In its expanded state, this hydrogel efficiently adsorbed Ag(I) ions. Experimental results indicated that the adsorption capacity of the thiol−ene−based hydrogel for Ag(I) ions strongly depended on pH, reaching 92.80 mg/g under strongly acidic conditions (pH = 0.5). Five consecutive −adsorption–desorption cycles were conducted using 0.8 M thiourea at pH 3, and no significant loss in adsorption capacity was observed. Thakshila et al. [48] also reported a novel acrylated thiourea−based polyethylene glycol diacrylate (ATU−PEGDA) hydrogel synthesized via the photo−reactive method, selectively recovering Ag(I) and Pd(II) from electrolytic wastewater, with a maximum adsorption capacity of 83.33 mg/g for Ag(I). Using 0.5 M thiourea and 0.001 M HNO_3_ as the eluent, the ATU−PEGDA hydrogel could be fully regenerated within 120 min, forming thiourea branched structures. Compared to the initial hydrogel, not only was the adsorption capacity enhanced, even after 10 consecutive −adsorption–desorption cycles, the adsorption capacity of ATU−PEGDA for Ag(I) could still reach 111.34 mg/g. Additionally, the color change of ATU−PEGDA hydrogel facilitates the determination of adsorption saturation and complete desorption of Ag(I).

Furthermore, chelating resins composed of two functional groups, chelating ions and a polymer matrix as a carrier, contain atoms with lone pairs of electrons in their functional groups, such as O, N, S, P, etc., capable of forming cross−linked structures with metal ions to form multi−coordination complexes. These resins exhibit advantages such as simple operation, low cost, and easy regeneration [49]. For example, Lin et al. [50] synthesized a novel chelating resin (TSC−CC) by reacting melamine with aminothiourea. The adsorbent exhibits excellent stability under acidic conditions, selectively adsorbing Ag(I) from acidic solutions with an adsorption capacity of 872.63 mg/g at an acid concentration of 0.5 mol/L. After three regeneration cycles, when TSC−CC was added to an initial Ag(I) solution with a concentration of 320 mg/L and acidity of 0.5 mol/L, the removal efficiency remained at 73.6%, demonstrating significant potential for practical applications. Additionally, in the pursuit of versatile applications of chelating resins in wastewater, Wang et al. [51] utilized submicron resorcinol−formaldehyde (RF) resin nanoparticles (NPs) to generate a brominated resin by combining them with bromine from halogen−containing wastewater, enabling cyclic recovery of silver in wastewater. Within 5 h, it exhibited a high capture capacity of 93.4 mg/g for the initial concentration of 100 ppm Ag(I). This not only achieves bromine removal but also reduces heavy metal pollution, realizing resin resource utilization. Chelating resins exhibit significant potential in the treatment and recovery of silver from different wastewater streams. Chelating resins also show great potential for the treatment and recovery of silver in different wastewaters. Nawaz et al. [52] prepared a thiol−group−functionalized ion exchange resin capable of directionally recovering silver from laundry wash liquids and successfully converting it into silver sulfide nanoparticles. In a 0.5 mg/L Ag(I) solution, it can process up to 14,000 bed volumes (BVs) with a removal efficiency of 84%. Simultaneously, using a regeneration solution of 152 BV, over 90% of the resin can be regenerated and recovered, with no significant loss in performance after more than five cycles of reuse.

To achieve selective separation of silver ions, the formation of binding sites with specific coordination is particularly crucial. Molecularly imprinted materials, as typical chelating coordination adsorbents, have been widely researched and utilized. Molecularly imprinted materials are polymer materials formed by the template effect of specific molecules. During the synthesis of molecularly imprinted materials, the target molecule is first chosen as the template and then polymerized with functional monomers or cross−linking agents to form a cavity structure with the shape and function of the target molecule. After polymerization, the template molecules are removed, leaving behind cavities that match the template molecules, forming molecularly imprinted materials with specific recognition properties [53]. Koray et al. [54] synthesized a silver ion−imprinted polyhydroxyethyl methacrylate (PHEMA)−based cryogel using N−methylacryloyl−L−cysteine as the functional monomer. The maximum adsorption capacity of the silver ion−imprinted polymer cryogel for Ag(I) was 49.27 mg/g. To investigate the affinity of the silver ion−imprinted PHEMAC cryogel column, the photosensitive film material was immersed in a nitric acid solution for Ag(I) recovery, achieving a recovery rate of 72.8%. To assess its reusability, cyclic experiments were conducted using a 0.1 M EDTA solution, and over 10 cycles of −adsorption–desorption were performed without significant loss. Huo et al. [55] employed surface molecular imprinting technology to prepare Ag(I) imprinted adsorbents for treating Ag(I)−contaminated wastewater, demonstrating higher affinity and selectivity for the imprinted ion (Ag(I)) than other non−imprinted metal ions. The prepared Ag(I) imprinted adsorbent exhibited a maximum adsorption capacity of 199.2 mg/g at an initial silver concentration of 1200 mg/L and an adsorbent dosage of 3.0 g/L. Moreover, easy desorption was achieved using a 0.1 M Na_2_S_2_O_3_ solution as the eluent.

In addition to hydrogels, molecularly imprinted materials, and chelating resins, Ding et al. [56] synthesized a novel linear phenanthroline−based polymer—_L_−PRL—via the chemical oxidative polymerization method, with a maximum adsorption capacity of 325.8 mg/g for Ag(I) at pH 0. After four consecutive regeneration cycles, l−PRL retained 93.5% of its initial adsorption capacity for Ag(I), and it could almost completely remove Ag(I) from low−concentration industrial wastewater containing silver. Moreover, Zhang et al. [57] reported the synthesis of a highly stable benzimidazole−linked polymer (BILP), whose rigid structure and open groups enable selective binding of silver ions and, upon reduction by NaBH_4_, silver nanoparticles are formed. Additionally, a significant amount of nitrogen−containing heterocycles and loaded elemental silver exhibit a synergistic adsorption effect on iodine vapor, offering new insights into the development of polymer adsorbent materials. Table 2 summarizes the adsorption performance of other polymer materials towards Ag(I).

## 3. Adsorption Mechanisms

**Figure 2:**
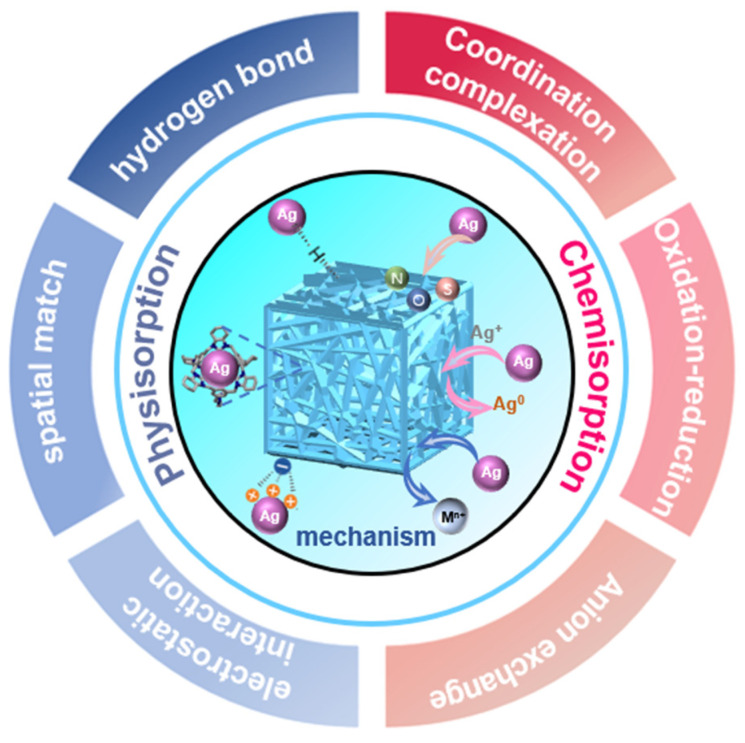
Adsorption mechanism of Ag(I) by different adsorbents.

As widely recognized, the adsorption mechanisms of adsorbents for Ag(I) in water mainly encompass chemical adsorption and physical adsorption [58]. As shown in Figure 2, chemical adsorption involves the formation of covalent or coordination bonds between surface functional groups or active sites and silver ions, where the electron donation from surface functional groups facilitates the formation of chemical bonds with silver ions [59]. Physical adsorption, on the other hand, relies on attractive forces (e.g., van der Waals forces, electrostatic forces) to adsorb Ag(I) onto the surface of the adsorbent, depending on non−covalent interactions between the adsorbent surface and Ag(I) [60]. These two adsorption mechanisms intertwine during the adsorption process, jointly determining the adsorption capacity and selectivity of the adsorbent for silver ions [61]. Moreover, solution conditions (such as pH value, temperature) and surface properties of the adsorbent also play critical roles in influencing the adsorption process. A profound understanding of the mechanisms underlying the adsorption of Ag(I) by adsorbents is of significant importance for optimizing adsorption systems, enhancing adsorption efficiency, and achieving cyclic regeneration.

### 3.1. Coordination Complexation

**Figure 3:**
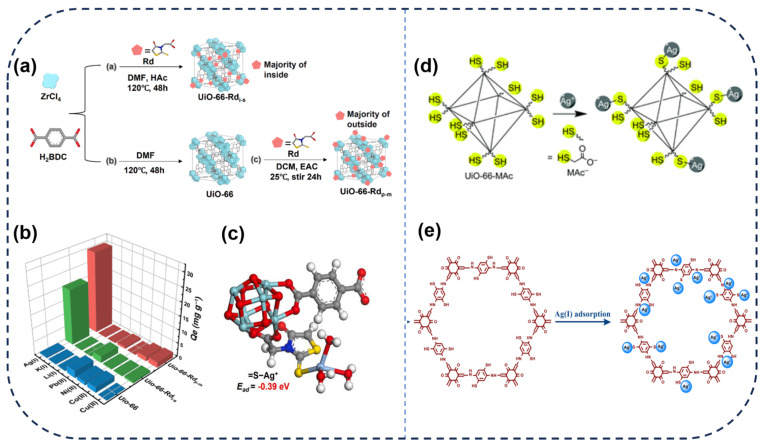
(**a**) Synthesis of UiO−66−Rd_i−s_ and UiO−66−Rd_p−m_. (**b**) Competitive adsorption of coexisting metal ions. (**c**) The optimized adsorption configurations and adsorption energy of Ag(I) on S sites. The adsoption mechanism of Ag(I) by (**d**) UiO−66−MAc. (**e**) COF−SH [4,20,65].

Coordination complexation refers to the process whereby Ag(I) in water forms coordination bonds with the adsorbent material. The soft−hard acid−base theory proposed in 1963 has played a crucial role in coordination complexation. Silver ions, characterized by relatively large atomic radii and low ionization energies, are typically classified as soft acids. They often form stable complexes with soft base ligands in coordination complexation [62,63]. Current research suggests that the adsorbents functionalized with atoms such as N, O, and S exhibit strong coordination ability with Ag(I) ions. [64]. For instance, adsorbents functionalized with sulfur−containing groups such as thiourea, thiol, and thioether show high selectivity for Ag(I) adsorption [4,65,66]. Increasing sulfur content enhances the adsorption capacity and selectivity for Ag(I). Two types of UiO−66 modified with rhodanine (Rd) prepared by the Ding et al. [20] exhibited significant adsorption effects on Ag(I) (Figure 3a,b). This is mainly attributed to the chelation coordination between Ag−S on Rd, and theoretical calculations suggest that the adsorption binding energy between Ag−S reaches up to −0.39 eV (Figure 3c). Furthermore, the energy distribution of the D−A isotherm model confirms that UiO−66−Rd_p−m_ possesses more effective Ag(I) adsorption sites compared to UiO−66−Rd_i−s_. The higher density of Rd outside UiO−66−Rd_p−m_, with a S/Ag ratio of 1.41, surpasses UiO−66−Rd_i−s_ (0.59), due to the multiple coordination of active S sites with Ag(I), significantly enhancing the adsorption rate and selectivity. Furthermore, Pan’s group synthesized COF−SH, which has enhanced selectivity for Ag(I) adsorption owing to its high sulfhydryl content (Figure 3e).

However, an increase in sulfur content may adversely affect the properties of the material. In recent years, it has been reported that mercaptoacetic acid is a suitable sulfur−containing thiol modifier because its structure is similar to that of acetic acid, making it applicable for introducing sulfur thiol groups in the one−pot synthesis of MOFs. Moll et al. [65] utilized mercaptoacetic acid (HMAc) as a modifier to introduce thiol groups into the UiO−66 framework through a simple and low−cost method. As shown in Figure 3d, the adsorption of Ag(I) by UiO−66−MAc mainly depends on its surface −SH functional groups. UiO−66−MAc−50 eq can achieve up to 7 w% high sulfur doping, with the maximum uptake of Ag(I) reaching 84 mg/g, while maintaining the porosity and surface area of UiO−66. However, the use of very high HMAc equivalents can cause UiO−66 to transition from the common face−centered cubic (fcu) topology to the rare hexagonal close−packed (hcp) topology. Moreover, as the amount of HMAc added increases, the pore size of UiO−66−Mac gradually decreases. The change in pore size results in UiO−66−Mac−100 eq having a maximum adsorption concentration of only 32 mg/g for Ag(I). Therefore, it is essential to adjust the sulfur doping level appropriately based on the intrinsic conditions of the adsorbent material without compromising its properties, which can enhance the adsorption capacity for Ag(I).

**Figure 4:**
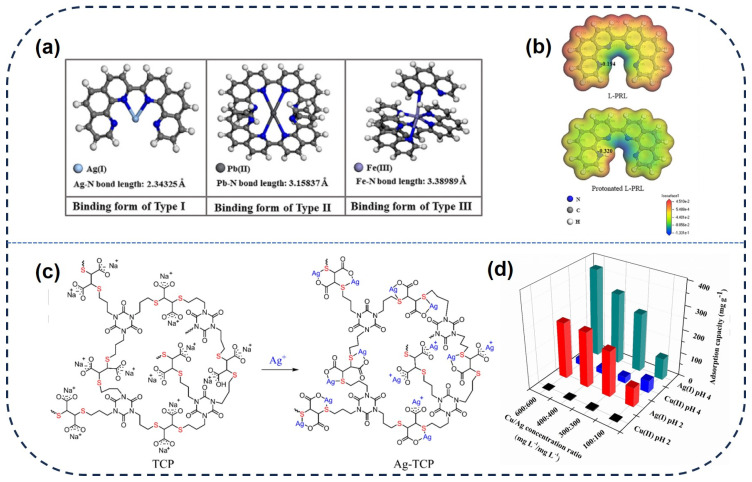
(**a**) The bond length of Ag−N, Pb−N, and Fe−N on _L_−PRL. (**b**) Atomic charges of _L_−PRL and protonated _L_−PRL by theoretical calculations. (**c**) The adsorption mechanism of Ag(I) by TCP. (**d**) Effect of metal ion concentrations on the competitive adsorption of Ag (I) and Cu (II) by TCP [56,68].

Meanwhile, nitrogen−rich functionalized adsorbents exhibit significant selective removal of Ag(I), with the self−polymerization of nitrogen−rich ligands being an ideal approach. Ding et al. [56] synthesized a novel linear o−phenanthroline−based polymer—_L_−PRL—with abundant nitrogen content and excellent stability. Theoretical calculations reveal that the nitrogen groups exhibit more negative electrostatic potentials compared to other regions, and for H^+^ protonated _L_−PRL, the electrostatic potential of nitrogen groups is more negative than that of _L_−PRL (Figure 4b). This provides strong theoretical support for enhancing the adsorption capacity of acidic−enhanced _L_−PRL and indicates that the adsorption sites coordinating with Ag(I) are nitrogen−containing groups on _L_−PRL. Furthermore, as shown in Figure 4a, compared to other ions, _L_−PRL exhibits the shortest Ag−N bond length with Ag(I) in water, forming the most stable complexes and enhancing selectivity to a certain extent. Additionally, _L_−PRL retains 93.5% of its adsorption capacity after four consecutive regeneration cycles, demonstrating superior recyclability and durability, making it applicable for treating low−concentration Ag(I)−contaminated wastewater.

Numerous studies demonstrate that synergistic adsorption among various functional groups enhances the adsorption capacity and selectivity for Ag(I) [67]. Huang et al. [68] synthesized a novel adsorbent (TCP) using the thiol−ene polymerization method. As shown in Figure 4c, TCP is a dense polymer with abundant thioether and carboxyl functional groups, and due to the synergistic effect between carboxyl and thioether groups, TCP significantly enhances the adsorption capacity for Ag(I). Regardless of whether the pH is 2 or 4, TCP exhibits outstanding adsorption selectivity for Ag(I), with a maximum adsorption value of 5.2 mmol/g at pH = 4 (Figure 4d).

### 3.2. Redox Reaction

**Figure 5:**
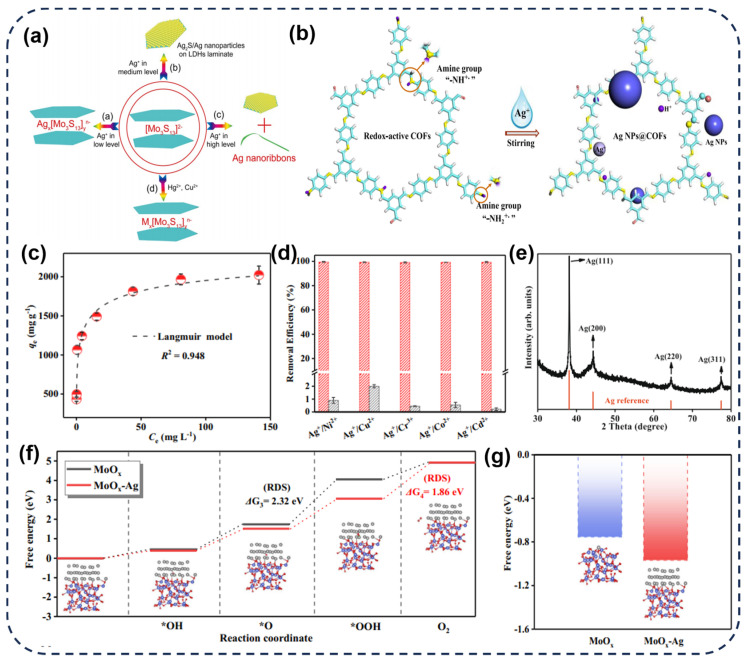
(**a**) Possible sorption mechanism during Mo_3_S_13_^−^ LDH adsorbing heavy metal ions of Ag(I), Cu(II), and Hg(II). (**b**) The reductive sorption mechanism of Ag(I) onto the COF−LZU1. (**c**) The adsorption capacity of MoO_x_ for Ag(I), (**d**) the adsorption selectivity, (**e**) and the XRD after adsorption of Ag(I). (**f**) Free energy of intermediary process of the reduction deposition for Ag(I) on MoO_x_ and MoO_x_−Ag (rate−determining step, RDS) (**g**) and Ag(I) reduction deposition on MoO_x_ and MoO_x_−Ag. The insets show the optimized MoO_x_ and intermediates on MoO_x_−Ag [21,70,71].

Redox reactions typically involve electron transfer and changes in the oxidation states of atoms [69]. Yang et al. [21] inserted Mo_3_S_13_^2−^ into MgAl−LDH to obtain a new material, Mo_3_S_13_^−^LDH, which not only exhibits excellent absorption and selectivity for heavy metal ions such as Cu(II), Hg(II), and Ag(I) but also has a high adsorption capacity of up to 1073 mg/g for Ag(I). Under conditions of high silver concentration, Mo_3_S_13_^−^ LDH successfully reduces Ag(I) to elemental silver (Figure 5a), and this material also possesses good extraction and separation capabilities for silver at other concentrations, making it promising for applications in practical water bodies. Additionally, many amine groups on covalent organic framework materials linked by imine bonds and rich unsaturated amine groups at the edges possess reducibility. Therefore, Wang et al. [70] synthesized an amine−based covalent organic framework with redox activity by condensing 1,3,5−triformylbenzene and p−phenylenediamine. Experimental results confirm that the N sites of the framework’s amine groups facilitate the reduction of Ag(I) to the metallic state (Figure 5a). Furthermore, the recovered Ag@COF material can further serve as a new adsorbent for removing Hg(II) ions from water, exhibiting ultra−high recycling capability. In some cases, adsorption and redox processes may occur simultaneously or interact with each other. Shao et al. [71] synthesized a mixed−valence molybdenum oxide (MoO_x_) targeted adsorbent with both Mo(V) and Mo(VI) using a simple electrochemical method. As shown in Figure 5c, it exhibits super−strong adsorption capacity for Ag(I), with an adsorption capacity as high as 2605.91 mg·g^−1^, accompanied by excellent adsorption selectivity (Figure 5d). Theoretical calculations and experimental results indicate that after capturing Ag(I), XRD reveals that Ag^0^ is significantly deposited on the adsorbent surface of MoO_x_ (Figure 5e). Density functional theory (DFT) calculations further reveal that MoO_x_ mainly captures Ag(I) through a self−enhanced reduction deposition mechanism. In summary, Mo(V) on MoO_x_ serves as an electron donor, reducing Ag(I) to metallic Ag^0^, while Ag^0^ on the surface of MoO_x_ reduces the energy barrier for Ag(I) reduction deposition, enhancing subsequent reduction processes (Figure 5e). Importantly, as shown in Figure 5f, the free energy of Ag(I) reduction on MoO_x_−Ag is lower than that on MoO_x_, indicating that Ag(I) is more easily reduced and deposited on MoO_x_−Ag, which is the reason for its high absorption of Ag(I).

### 3.3. Ionic Exchange

Ionic exchange is a method whereby materials possessing specific functional groups undergo exchange reactions with silver ions in aqueous solutions. Compared to other methods, this approach is particularly effective for the treatment of trace or ultratrace metal ions in wastewater and typically involves resins with high stability and acid−base resistance [72]. For instance, the ion exchange resin TSC−CC, as mentioned above [50], employs –N–C=S and –NH_2_ functional groups on its monomers to chelate Ag(I) and undergo ion exchange with charged groups from Ag(NO_3_)^2−^. Atia et al. [73] immobilized various functional groups onto methyl methacrylate−glycidyl methacrylate/divinylbenzene resin, where in the presence of hydrochloric and nitric acids, the uptake of Ag(I) was attributed to the formation of anionic Ag(Cl)^2−^ and Ag(NO_3_)^2−^. These anions exchange with the conjugate anions on the resin surface, resulting in a maximum uptake capacity of 308.51 mg/g for Ag(I).

Additionally, the adsorption process using inorganic materials often involves an ionic exchange mechanism. For instance, Liu et al. [74] synthesized a novel three−dimensional −core–shell structured magnetic Fe_3_O_4_@SiO_2_@CaSiO_3_ composite material via precipitation. The experimental results showed that after adsorbing Ag(I), Ca^2+^ was released from Fe_3_O_4_@SiO_2_@CaSiO_3_, accounting for approximately 13.80–16.89 mol% of the adsorption. Ag(I) was then exchanged with Ca^2+^ in the layered CaSiO_3_ on Fe_3_O_4_@SiO_2_@CaSiO_3_ to form low−solubility silicates, resulting in an adsorption capacity of 127.84 mg/g at equilibrium. Furthermore, this composite material exhibits excellent magnetic separation performance and recyclability, enabling continuous wastewater treatment.

### 3.4. Physical Adsorption

In addition to chemical adsorption, physical adsorption plays a crucial role throughout the adsorption process. Firstly, electrostatic interactions, based on charge interactions, are significant. In this process, Ag(I) in water is attracted to adsorbent materials with opposite surface charges. The zeta potential, which reflects the surface charge of adsorbents in aqueous solutions under different pH conditions [75], is a critical factor influencing electrostatic interactions. Huang et al. [76] synthesized chitosan−coated magnetic silica −core–shell nanoparticles (FO@SD@CS), which exhibited pH−responsive adsorption behavior towards Ag(I), with the adsorption capacity increasing with pH. At a lower pH, protonation of the surface amino and hydroxyl groups on FO@SD@CS occurs, leading to competition with Ag(I) for adsorption sites due to increased H^+^ concentration. At pH 6.0, the selective adsorption capacity reached 85.86 mg/g. Furthermore, the adsorption of Ag(I) in composite wastewater containing multiple metal ions and dyes was investigated. It was found that electrostatic attraction exists between the material and methylene blue (MB) molecules, enhancing the adsorption of Ag(I) in the dye−–metal binary system. The presence of MB enhances the adsorption capacity of FO@SD@CS for Ag(I) from 116.28 mg/g to 156.25 mg/g, demonstrating synergistic adsorption.

Moreover, cavity matching refers to the adaptability between the micro−pores or cavities in adsorbents and the size and shape of adsorbed molecules. The internal cavities of adsorbents provide significant advantages when adsorbing specific ions [77]. For instance, Fan et al. [78] utilized Ag(I) as the imprint ion (Ag−TCM) and achieved selective adsorption of Ag(I) through a surface thiourea−–chitosan coating. At pH 5, the maximum adsorption capacity of Ag−TCM for Ag(I) was 4.93 mmol/g. Membranes serve as physical barriers, and separation based on membrane pore size is one of the primary mechanisms. Typically, solutes smaller than the membrane pore size pass through the membrane, while larger solutes are retained on the other side. Yin et al. [79] prepared composite 3−allylrodonine−modified 3−AR/PVDF membranes using a nonsolvent−induced phase separation method, exhibiting hydrophilic surfaces and uniform porous structures with an average pore size of 47.03 nm. The 3−AR/PVDF−0.130 membrane demonstrated excellent performance in terms of permeability, selectivity, and retention of Ag(I), achieving an equilibrium adsorption capacity of 45.15 mg·cm^−2^ when the membrane thickness was 0.16 mm.

Hydrogen bonding, an important non−covalent interaction between molecules or within molecules, differs from van der Waals forces in that it is saturated and directional, with stronger intermolecular forces. While hydrogen bonding in the adsorption of heavy metals may not be as significant as other types of chemical bonds or interactions, it can play a role in specific cases. Previous studies have shown that hydrogen bonding between heavy metal ions and adsorbents can significantly enhance the adsorption performance of the adsorbent [80]. The newly designed adsorbent ZMC−MAH−TEPA by Liu et al. [81] may interact with Ag(I) through hydrogen bonding, leading to improved adsorption capacities for both Ag(I) and Cr(VI) compared to the original adsorbent. Additionally, CB−G3 prepared by Liu et al. [41] with branched internal molecular cavities can encapsulate Ag(I) through weaker hydrogen bonding interactions, enhancing the adsorption performance of the adsorbent itself.

## 4. Defects and Challenges of Adsorbents

Although adsorbents offer advantages in treating Ag(I) in water compared to other methods, there are potential environmental issues associated with their preparation process. For example, the use of toxic organic solvents (such as DMF, dichloromethane, etc.) in the synthesis of novel adsorbent materials (e.g., MOFs, COFs, etc.) via solvothermal methods may pose potential hazards to the environment and human health when released into the air.

The stability of adsorbents is also a key issue, as the adsorption capacity of most reported adsorbents tends to decrease with increasing acidity due to the breakdown of the adsorbent structure induced by strong acids. Generally, some adsorbents exhibit long−term operational stability under neutral or near−neutral conditions. However, under harsh conditions (e.g., strong acid or alkali conditions), adsorbents may partially or completely lose the integrity of their frameworks or evolve into amorphous structures with or without porosity. Additionally, temperature can also affect the stability of adsorbents, as some adsorbents may undergo thermal decomposition at high temperatures, leading to structural damage or deactivation. This may be attributed to internal bond breakage or changes in molecular structure. Therefore, it is necessary to evaluate the thermal decomposition characteristics of adsorbents at high temperatures to avoid irreversible structural damage during processes.

The physical properties of adsorbents themselves also influence their adsorption performance. Insufficient surface area of adsorbents may result in inadequate adsorption sites, affecting adsorption capacity. Moreover, adsorbents typically contain pore structures, including micropores, mesopores, and macropores. If the pore structure is unreasonable or the pore distribution is uneven, diffusion limitations may occur, affecting adsorption rates and efficiency, as well as subsequent recovery and regeneration of adsorption. For example, the pore structure of ion exchange resins is prone to contamination and requires cumbersome processes for functional regeneration, with high demands for elution solvents. Consequently, the process becomes complex and costly, making it difficult to be widely applied in practical industrial applications. Moreover, over time, adsorption sites on adsorbents may gradually saturate, limiting the adsorption capacity of the adsorbent. This leads to decreased efficiency of adsorbents in treating high−concentration silver−containing wastewater, necessitating frequent replacement or regeneration operations. Furthermore, some adsorbents may be difficult to recover and regenerate effectively after adsorption, requiring costly recovery methods, which may cause secondary pollution during the recovery process. This increases the cost of wastewater treatment and negatively impacts environmental sustainability.

Additionally, the surface functional groups of adsorbents may have an affinity for multiple ions, and their selectivity may be influenced by other components in wastewater. This may result in poor selectivity for Ag(I) when adsorbents are used to treat complex wastewater compositions. Xie et al. [82] constructed a new material, poly pyrrole/MoS_4_^2−^ (MoS_4_−Ppy), which exhibited high acid stability and good absorption capacity for heavy metal ions such as Hg(II), Ag(I), Cu(II), and Pb(II). Although MoS_4_−Ppy showed significant selectivity for Ag(I) over Cu(II) under weakly acidic and strongly acidic conditions, it exhibited efficient removal of both Ag(I) and Hg, which have affinity for sulfur, with removal efficiencies exceeding 99.9% at pH ≈ 1, making effective separation difficult.

## 5. Adsorbent Optimization

To address the aforementioned challenges, optimization of traditional adsorbents is necessary. This optimization can involve enhancing the adsorption capacity, selectivity, and regenerability of adsorbents to meet different application requirements. Concurrently, the recyclability of adsorbents is crucial for sustainability and economic viability.

### 5.1. Modification of Adsorbents

Adsorbent modification is a process involving the introduction of external substances or changes in the adsorbent structure to enhance its adsorption performance or impart specific functionalities [83,84,85]. Membrane separation technology has been reported for the separation and recovery of Ag(I), overcoming the limitations of traditional membrane separation methods, which rely solely on pore size and electrostatic repulsion for ion separation. This approach can enhance the selectivity of the adsorbent for Ag(I) and improve separation efficiency, achieving a synergistic effect [86]. Shawky et al. [87] innovatively introduced ion−imprinting technology into membranes to synthesize a novel ion−imprinted membrane for the selective removal and pre−concentration of Ag(I) from aqueous solutions. Specifically, a new ion−imprinted membrane was synthesized by cross−linking chitosan (CS) and polyvinyl alcohol (PVA) with glutaraldehyde (GA) as the cross−linking agent. The competitive removal capacity of the imprinted membrane for Ag(I) was higher than that of the non−imprinted membrane. The ion−imprinted membrane exhibited excellent selectivity for silver in matrices containing interfering ions (such as Cu^2+^ and Ni^2+^). Moreover, the ion−imprinted membrane demonstrated good reusability and stability, maintaining a removal rate of not less than 85% after five cycles of reuse.

Furthermore, magnetic modified nanomaterials, due to their high adsorption efficiency, ease of magnetic separation, and reusability advantages, are widely used in the adsorption of heavy metal ions [88]. These adsorbents are distinguished by a magnetic core primarily constituted of materials such as iron, nickel, or cobalt. The presence of this magnetic core confers responsiveness to the adsorbent when subjected to an external magnetic field, enabling swift mobility and precise localization guided by the magnetic field. Concurrently, the surface of the magnetic adsorbent is enveloped with an adsorbent outer layer, responsible for interacting with the target substance through adsorption. Typically, these outer layers of the adsorbent possess a high specific surface area and specific chemical functional groups, thereby facilitating the effective adsorption of the target substance. Notably, magnetite, endowed with magnetic separation properties, has been engineered for the removal of metal ions, thereby facilitating recycling and averting secondary pollution. Liu et al. [4] synthesized a novel grafted zeolite/Fe_3_O_4_/chitosan (ZMC−MAH−TEPA) adsorbent through organic−–inorganic chemical cross−linking to enhance the chemical stability of chitosan. The adsorbent possesses abundant −NH_2_ and −NH− groups, with a maximum adsorption capacity for Ag(I) of 70.12 mg/g at pH 5.

To be highly applicable in environmental and industrial applications and achieve large−scale production, transforming powder materials into or attaching them to large−volume solids is also a popular method of adsorbent modification. Wang et al. [89] directly prepared a series of dual−functional polysiloxane microspheres—ASPSS—with adjustable porous structures and functional group contents via the sol−gel method. The synergistic effect of amino and sulfur groups on ASPSS microspheres exhibited excellent adsorption performance for Ag(I) at room temperature, with an adsorption capacity of 3.86 mmol/g, and the ASPSS microspheres also showed good regenerability, maintaining a regeneration rate of 91.43% after five −adsorption–desorption cycles, demonstrating promising practical application value. Alsulami et al. [90] synthesized a microporous functionalized silica polymer sponge composite material using a triblock copolymer surfactant. The synthesized SiO_2_@TF exhibited outstanding adsorption capacity for Ag(I), with a maximum adsorption capacity of 1356 mg/g, and could be applied in practical waters such as drinking water, hospital wastewater, and seawater. Shen et al. [39] immobilized chitosan on porous glass beads, achieving an adsorption capacity of 5.15 mmol/g for Ag(I). The adsorbent used maintained its adsorption capacity after five cycles of adsorption, indicating high mechanical strength, large specific surface area, and non−expansiveness in water, providing possibilities for practical applications.

**Figure 6:**
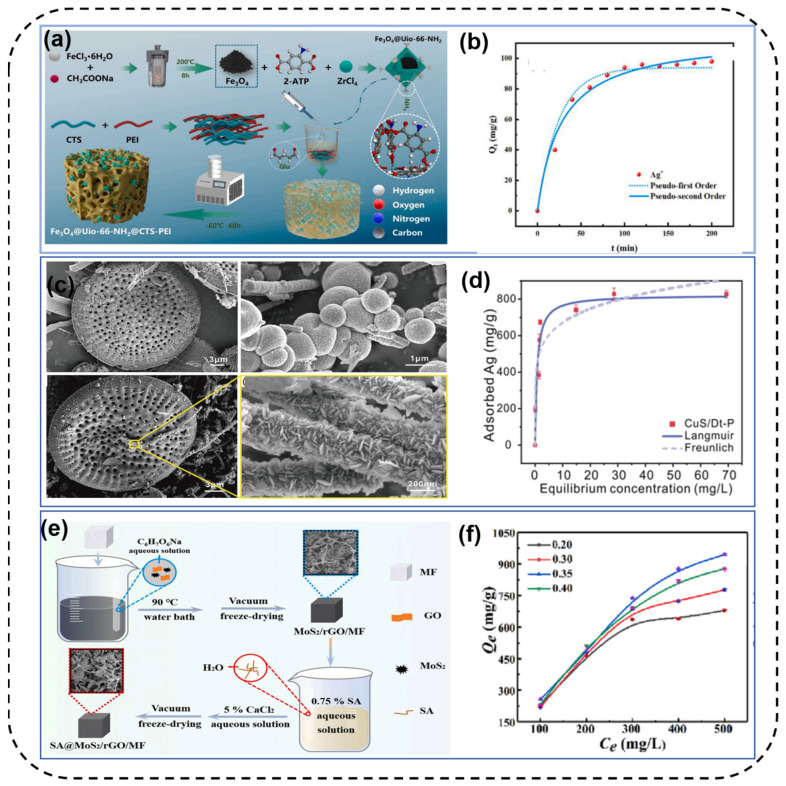
(**a**) Schematic illustration of synthetic route for Fe_3_O_4_@UiO−66−NH_2_/CTS−PEI hydrogel. (**b**) (**c**) The adsorption kinetics for Ag(I) on Fe_3_O_4_@UiO−66−NH_2_/CTS−PEI hydrogel. (**c**) SEM images for surface morphologies of Dt (upper left); CuS nanoparticles (upper right); and CuS/Dt−P (low right and left). (**d**) Adsorption isotherm of CuS/Dt−P for Ag(I). (**e**) Fabrication of the SA@MoS_2_/rGO/MF and (**f**) effect of MoS_2_ addition amount on the adsorption performance of MoS_2_/rGO/MF [91,92,93].

Composite materials combining various adsorbents are also an ideal way to treat silver−containing wastewater, overcoming the shortcomings of single adsorbents. The synergistic effect of different functional groups on different adsorbents also plays a significant role in the adsorption of Ag(I), enhancing the adsorption capacity and selectivity. Zheng et al. [91] successfully prepared a novel magnetic hydrogel composite material (Figure 6a) by embedding MOF composites (Fe_3_O_4_@UiO−66−NH_2_) into CTS−PEI hydrogel. The introduction of PEI and Fe_3_O_4_@UiO−66−NH_2_ provided numerous N−containing adsorption sites, and the adsorption performance of the composite material was significantly higher than that of single materials over a wide pH range, reaching adsorption equilibrium within 200 min, with a maximum adsorption capacity of 226.88 mg/g (Figure 6b). The adsorbed composite material could also be converted into silver−doped photocatalysts through reduction reactions, showing promising efficiency in rhodamine degradation and further resource utilization. Yuan et al. [92] prepared a porous CuS/modified diatomite composite material using a mild −in situ coating process. In summary, a uniform coating of copper sulfide nanosheets on pre−modified diatomite was achieved, retaining the porosity of the diatomite carrier. Compared to pure copper sulfide nanoparticles, the composite material exhibited a wider distribution of pores, higher specific surface area, and larger pore volume (Figure 6c). Figure 6d indicates a maximum adsorption capacity of 822.7 mg/g for Ag(I), far exceeding that of raw diatomite (9.87 mg/g), unmodified composite material (465.2 mg/g), and pure copper sulfide nanoparticles (635.5 mg/g). Yang et al. [93] incorporated MoS_2_ into a three−dimensional (3D) reduced graphene oxide (rGO) system in one step, simultaneously loading it into melamine foam (MF) with a 3D network structure, and then constructing an alginate (SA) cross−linked network through Ca^2+^. This resulted in a separable and structurally stable three−dimensional network adsorbent (SA@MoS_2_/rGO/MF) (Figure 6e). To expose as many adsorption sites as possible and provide as many channels as possible for ion transport, Figure 6f demonstrates the importance of MoS_2_ addition and SA concentration on the adsorption performance of Ag(I). The optimal composite material achieved a high adsorption capacity of 1012.51 mg/g for Ag(I).

### 5.2. Regeneration and Recycling of Adsorbents

To overcome the constraints of traditional adsorption processes in the current recovery and treatment of Ag(I) and promote green and sustainable development, the regeneration capability of adsorbents provides a solid foundation for the extraction of Ag(I). Generally, the regeneration process of adsorbents includes desorption, target ion collection, washing, regeneration, and drying. The significance of desorbing agents is self−evident [94,95]. For instance, hydrochloric acid (HCl) or nitric acid (HNO_3_) is commonly used to desorb silver from adsorbents. These acidic desorbing agents effectively dissolve silver ions and alter the surface charge characteristics of the adsorbent during the desorption process, facilitating the desorption of silver ions from the adsorbent. Yao et al. [96] utilized 1 mol/L HNO_3_ as the desorbing agent to separate Ag(I) from AC−SH adsorbent. After three consecutive −adsorption–desorption cycles, the adsorption capacity of Ag(I) on AC−SH−80 remained at 96.7% of the initial adsorption capacity. Nevertheless, in comparison to previous instances, there will be a notable decrease in the selectivity of the adsorbent. Simultaneously, under acidic conditions, thiourea forms strong cationic complexes with Ag(I). Zhao et al. [97] selected a mixed solution of 0.1 mol/L HCl and 1% thiourea as the desorbing agent. After six cycles of recovery, although incomplete recovery still existed during the desorption process, the removal efficiency of the PSDT adsorbent for Ag(I) remained at 89.9%; however, incomplete recovery remains prevalent during the desorption process.

**Figure 7:**
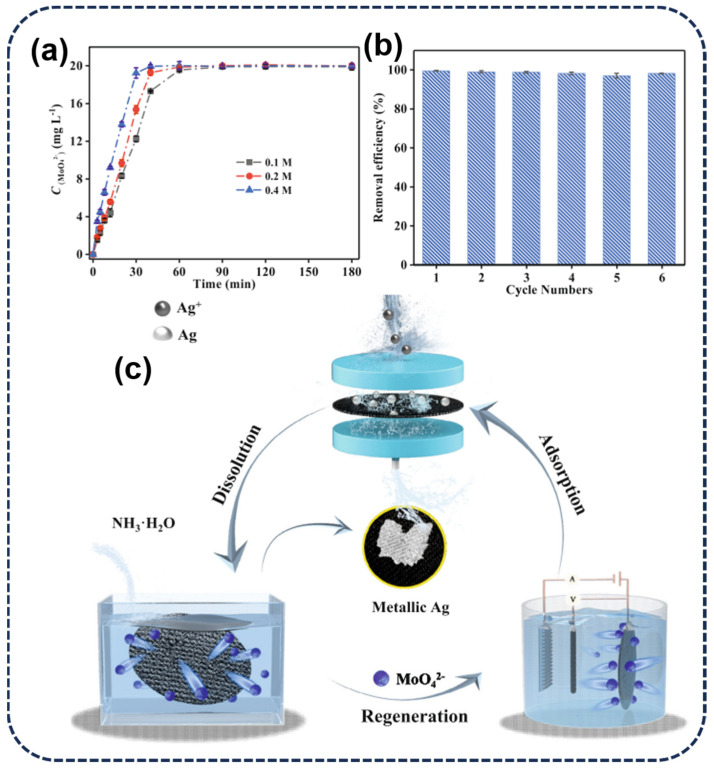
(**a**) Recovery of MoO_4_^2−^ using different concentrations of NH_3_·H_2_O (0.1–0.4 M). (**b**) Removal efficiency of the regenerated amorphous MoO_x_ for Ag(I). (**c**) Schematics of closed−loop recovery of metallic Ag [71].

In some cases, alkaline−desorbing agents such as NaOH or NH_3_·H_2_O can also be used to desorb silver from adsorbents. This type of desorbing agent changes the chemical form of silver by increasing the pH of the solution, facilitating its desorption from the adsorbent. Hence, in order to surmount the limitations inherent in conventional adsorption processes, Shao et al. [71] studied and designed a flow−through adsorption device, constructing a closed−loop recycling technology system for the recovery of Ag(I) from actual silver−plated wastewater and the regeneration of MoO_x_ (Figure 7c). Specifically, a diluted ammonia solution was used as the desorbing agent, enabling the recovery of silver and the regeneration of amorphous MoO_x_, with the recovery rate increasing with the concentration of NH_3_·H_2_O (Figure 7a). Moreover, after six cycles, the recovery rate remained at 97.1% (Figure 7b), breaking through the inherent bottleneck of traditional open−loop −adsorption–desorption technology and providing a new strategy for the selective capture and directional resource utilization of Ag(I) in complex and stringent silver−containing wastewater. On the other hand, the multiple applications of adsorbed adsorbents also represent a novel approach to green recycling. To implement the concept of 3Rs (Reduce, Reuse, Recycle), Ren et al. [98] proposed the formation of a high−value−added Ag/C/TiO_2_ photocatalyst by further calcining NH_2_−MIL−125 after adsorbing Ag(I), achieving photocatalytic degradation of organic pollutants such as methylene blue (MB) and phenol. The adsorbed material MIL−127/PoPD@Ag reported by Xue et al. [26] contains silver ions and metallic silver, making it an attractive antimicrobial agent. Additionally, composite materials can release a large amount of active silver through alkaline hydrolysis. They exhibit significant antibacterial ability against both Gram−negative and Gram−positive bacteria, showing great potential in clinical applications.

### 5.3. Selectivity of Adsorbents

In the treatment of industrial wastewater containing silver, there are often various coexisting metal ions such as copper, cadmium, cobalt, nickel, lead, and zinc, whose concentrations are typically much higher than that of Ag(I). Therefore, achieving selective adsorption or recovery of silver from such wastewater is crucial. A fundamental approach to enhancing the selectivity of adsorbents for silver ions involves customized material design. Functionalization of adsorbents to increase their affinity for silver ions, thus enabling preferential adsorption in complex matrices, has become a common strategy. For instance, some studies have demonstrated the strong affinity of silver ions by Rhodanine (Rd). Ding et al. [99] significantly enhanced the selectivity by grafting Rhodanine functional groups onto two metal-organic frameworks (UiO−66 and UiO−66−NH_2_), achieving maximum selectivity coefficients of UiO−66−Rd (16,586) and UiO−66−NH_2_−Rd (40,999), which are 727 and 1795 times higher than UiO−66, respectively. Moreover, Ding et al. [20] achieved a maximum selectivity coefficient of 1.7 × 10^4^ for UiO−66−Rd_p−m_ through post−modification, far exceeding the standard for evaluating selective adsorption capacity (10^4^).

On the other hand, utilizing the synergistic effects among different components of composite adsorbents or introducing selective recognition sites can further enhance the specificity for silver ions. For example, Huang et al. [76] utilized methylene blue (MB) in solution to occupy active sites on the FO@SD@CS surface, where MB molecules provide additional −SO_3_^−^ sources, potentially offering new active sites for capturing Ag(I) and thereby enhancing the selective removal capability in binary systems. Zeng et al. [100] investigated the adsorption of Ag(I) in solution by SiO_2_−supported nano−ferrioxalate (SNFO) composite materials synthesized from biotite minerals. The results indicate that the removal mechanism of Ag(I) is attributed to synergistic reduction interactions between ferrioxalate anions and ferrous ions. Even at low Ag(I) concentrations, the composite material exhibited high selective removal capability. In binary coexisting solutions of Ni−Ag, Co−Ag, Zn−Ag, Cd−Ag, and Cu−Ag, although the chelation between oxalate ions and interfering ions strengthened, thus weakening the removal efficiency of Ag(I), the removal efficiency of coexisting ions was almost zero, indicating excellent selectivity of the SNFO composite material for removing Ag(I) from the solution.

**Table 1:** Adsorption properties of several adsorbents for Ag(I).

Type	Adsorbent	Adsorption Capacity/(mg·g^−1^)	Equilibrim Adsorption/(min)	Adsorption Mechanism	Ref.
MOFs	(fcu) UiO−66 (hcp) UiO−66	84 36	120	Coordination −complexation	[66]
	NH2−MIL−125	192.5	60	Electrostatic −interaction/Coordination −complexation	[98]
	UiO−66−Rd UiO−66−NH_2_−Rd	112 163	15	Coordination −complexation	[99]
	Fe_3_O_4_@UiO−66−NH_2_/CTS−PEI	226.88	120	Coordination −complexation	[92]
	UiO−66−Rd_p−m_ UiO−66−Rd_i−s_	120 109	10 30	Coordination −complexation	[20]
	MIL−127/26PoPD	560	5	Redox −reaction/Coordination −complexation	[26]
COFs	COF−SH	609.89	16	Ionic −exchange/Coordination −complexation	[4]
	COF−HNU27	349.6	2	Coordination −complexation	[28]
	TAPA−BTDC	398.61	30	Coordination− complexation	[29]
	COF−LZU1	175	720	Redox −reaction	[70]
TMDs	MoS_2_–HNR	1350	60	Redox −reaction/Coordination −complexation	[101]
	MoS_4_−Ppy	480 (pH ≈ 5)/725 (pH ≈ 1)	5	Coordination− complexation/Redox −reaction	[82]
	SA@MoS_2_/rGO/MF	1012.51	1440	Redox −reaction/Coordination −complexation	[94]
	Mo_3_S_13_− LDH	1073	60	Redox− reaction/Coordination −complexation	[21]
	MoS_2_/MWCNTs	601.97	1440	Electrostatic −interaction/Coordination− complexation	[32]
Metal Oxide	SNFO	223.68	120	Redox− reaction Electrostatic interaction	[100]
	Fe_3_O_4_@SiO_2_@CaSiO_3_	127.84	150	Ionic− exchange/Coordination −complexation	[74]
	SiO_2_@TF	1356	20	Coordination −complexation	[90]
	ASPSS	416.37	240	Coordination− complexation	[89]
	MI−SiNSs MBI−SiNSs MTT−SiNSs	70.3 103.2 139.5	30	Coordination −complexation	[36]
	FO@SD@CS	116.28	200	Electrostatic −interaction/Coordination −complexation	[76]
	HMDNC	81.97	30	Electrostatic −interaction	[37]
	MoO_x_	2605.91	120/	Redox −reaction	[71]
Biomass Adsorbent	ZMC−MAH−TEPA	70.12	60	Electrostatic −interaction/Hydrogen −bonding/Coordination −complexation	[81]
	CB−G3	105.62	210	Electrostatic −interaction/Hydrogen −bonding/Coordination −complexation	[41]
	Chitosan supported on porous glass beads	555.52	90	Coordination− complexation	[91]
	DMC	1175.8	60	Coordination− complexation	[43]
	CuS/Dt−P	822.7	10	Coordination −complexation	[93]
	Eco−friendly adsorbent	23.92	90	Coordination− complexation	[102]

**Table 2:** Adsorption properties of other macromolecule polymer for Ag(I).

Adsorbent	Adsorption Capacity/(mg·g^−1^)	Equilibrim Adsorption/(min)	Adsorption Mechanism	Ref.
CAT	42.12	2160	Electrostatic −interaction/Coordination −complexation	[103]
P(Penta3MP4/PEGDA/AAc)	92.8	960	Coordination −complexation	[47]
P(Penta3MP4/PEG−DA/HEMA)	102	1440	Coordination− complexation	[104]
ATU−PEGDA	83.33	240	Electrostatic −interaction/Coordination− complexation	[48]
TSC−CC	872.63	500	Ionic −exchange/Coordination −complexation	[50]
PS−TMT	187.1	360	Coordination −complexation	[105]
BA−PGMA	157.05	300	Electrostatic −interaction/Coordination− complexation	[106]
T−PGMA	217.17	840	Coordination −complexation	[107]
Ag^+^−imprinted PHEMAC cryogel	49.27	90	Cavity match	[54]
Ag(I)−MGOIIP	77.6	10	Cavity match	[108]
ITG−OCMC	156.32	2880	Coordination− complexation	[87]
Mag−IIP	28.2	5	Cavity match	[109]
Ag^+^−imprinted poly (HEMA−MAC) polymeric nanoparticles	196.9	40	Cavity match/Coordination −complexation	[110]
Fe_3_O_4_@SiO2@TiO_2_−IIP	35.475	80	Redox −reaction/Cavity match	[111]
Ag−TCM	566	50	Cavity match	[78]
IIM	212	120	Coordination −complexation	[112]
Ag(I)−MIIP	62.5	15	Cavity match	[113]
Fe_3_O_4_@SiO_2_@Ag−IIP	149	30	Cavity match	[114]
MPHCP P−MPHCP	321 353	15	Coordination−complexation	[115]
_L_−PRL	325.8	20	Coordination −complexation	[56]
PSDT	303	300	Coordination −complexation	[96]
TCP	556	300	Coordination− complexation	[68]
P−2AT	336.98	90	Coordination −complexation	[116]
HOM−Si−MTHL	179.23	20	Coordination −complexation	[117]
Atrz	94	240	Coordination −complexation	[118]

Furthermore, employing advanced characterization techniques, including spectroscopic analysis, specific surface area measurement, and surface charge analysis, is crucial for evaluating the selectivity of adsorbents for silver ions. These techniques provide valuable information on the structure and chemical properties of adsorbents, aiding in the optimization of material design to achieve enhanced selectivity for silver ions.

### 5.4. Large−Scale Application of Adsorbents

The large−scale application of adsorbents for silver ion removal from industrial wastewater represents a significant research direction in the fields of environmental protection and resource recovery. To achieve industrial relevance, adsorbent selection must prioritize both high efficiency and stability within complex industrial water matrices. Given the typically elevated acidity of industrial wastewater, the preparation of materials with strong acid stability is paramount for practical applications. For instance, Pan et al. [4] demonstrated that the covalent organic framework material (COF−SH) retains excellent Ag(I) removal capabilities even in the presence of competing ions at concentrations ten times higher than Ag(I) in 1 M HNO_3_.

Furthermore, the ability of adsorbent materials to efficiently adsorb silver ions in complex wastewater matrices without interference from other components is a necessary condition for large−scale applications. For example, Xue et al. [26] reported on the composite metal-organic framework material (MIL−127/26 PoPD), demonstrating notable selectivity for extracting Ag(I) ions from seawater obtained from two distinct geographical regions, as well as from leachates derived from sensor contact points and waste photographic fixer solutions. Wu et al. [119] utilized the conductive polymer Poly(N−phenylglycine) (PNPG) to prepare a novel PNPG membrane via vacuum filtration for Ag(I) recovery from two practical solutions (waste PCB leachate and municipal solid waste incineration ash digestion solution). Despite the harsh environments of the actual solutions, PNPG still adsorbed approximately 96% of Ag(I).

Moreover, in large−scale applications, regeneration and recovery of adsorbents are also crucial. Effective regeneration processes desorb adsorbed silver ions from the adsorbent, restoring its adsorption performance for repeated use. Therefore, the regenerative properties of adsorbents should be carefully considered. For example, Huang et al. [120] developed a novel polyvinylidene fluoride (PVDF)−based composite membrane (PVDF/BDBTU) for Ag(I) adsorption by blending PVDF with N−benzoyl−N′,N′−dibutyl thiourea (BDBTU). After ten −adsorption–desorption cycles, no significant loss of adsorption capacity was observed, indicating strong reusability.

The practical large−scale application of industrial operations emphasizes economic efficiency, and low material development costs have always been pursued. Yao et al. [96] prepared activated carbon from waste cigarette butts and grafted it with thiol groups for Ag(I) adsorption, exhibiting high adsorption capacity (719.2 mg/g) and significant selectivity. The column experiments showed excellent processing capacity of 10,303 bed volumes (BVs) when the Ag(I) concentration in the solution was 10 mg/L. According to market surveys and synthesis yields, the material preparation cost was only USD2.5 × 10^3^/t, significantly lower than other adsorbents, demonstrating strong potential for practical applications. Additionally, Biswas et al. [43] proposed a simpler recovery technique than those used in commercial operations. Ag(I) was recovered in elemental form by incineration without the use of toxic eluents or any reducing agents. Extraction rates of Ag from actual waste liquids reached up to 99%, indicating the potential for large−scale applications of this process.

## 6. Outlook

Silver, as a pollutant in aquatic environments, possesses dual attributes of environmental resources, making the development of novel adsorbents for treating wastewater containing Ag(I) a topic of continued interest. This study extensively investigates the current status of various novel adsorbent materials in Ag(I) treatment in wastewater and provides a comprehensive analysis of their adsorption mechanisms. Given the existing deficiencies in current adsorbents, we emphasize the potential to mitigate these shortcomings through ingenious design and precise control.

To meet the demand for treating wastewater containing Ag(I), future research directions should focus on several key aspects:

(1) Addressing the existing deficiencies and issues of current adsorbents through systematic engineering optimization efforts aimed at enhancing their stability and sustainability. Additionally, successful translation of laboratory research outcomes into large−scale synthesis of adsorbents is crucial for advancing adsorption technology in wastewater treatment.
(2) Considering the pursuit of environmental friendliness, future adsorbent design should prioritize renewability and biocompatibility. Exploration of natural biological materials or biomimetic materials could lead to more environmentally friendly and sustainable adsorption technologies.
(3) On the theoretical front, the application of machine learning and artificial intelligence techniques holds promise in expediting the adsorbent design process. Analysis of extensive experimental data and simulation results can unveil more subtle adsorption patterns and optimization pathways, thereby providing deeper guidance for the precise design of adsorbents.

In summary, the future application of adsorbents in silver recovery from water may evolve towards more intelligent, environmentally friendly, and sustainable directions, offering innovative and efficient solutions for addressing water pollution issues.

## Data Availability

Not applicable.
